# Early management of isolated severe traumatic brain injury patients in a hospital without neurosurgical capabilities: a consensus and clinical recommendations of the World Society of Emergency Surgery (WSES)

**DOI:** 10.1186/s13017-022-00468-2

**Published:** 2023-01-09

**Authors:** Edoardo Picetti, Fausto Catena, Fikri Abu-Zidan, Luca Ansaloni, Rocco A. Armonda, Miklosh Bala, Zsolt J. Balogh, Alessandro Bertuccio, Walt L. Biffl, Pierre Bouzat, Andras Buki, Davide Cerasti, Randall M. Chesnut, Giuseppe Citerio, Federico Coccolini, Raul Coimbra, Carlo Coniglio, Enrico Fainardi, Deepak Gupta, Jennifer M. Gurney, Gregory W. J. Hawryluk, Raimund Helbok, Peter J. A. Hutchinson, Corrado Iaccarino, Angelos Kolias, Ronald W. Maier, Matthew J. Martin, Geert Meyfroidt, David O. Okonkwo, Frank Rasulo, Sandro Rizoli, Andres Rubiano, Juan Sahuquillo, Valerie G. Sams, Franco Servadei, Deepak Sharma, Lori Shutter, Philip F. Stahel, Fabio S. Taccone, Andrew Udy, Tommaso Zoerle, Vanni Agnoletti, Francesca Bravi, Belinda De Simone, Yoram Kluger, Costanza Martino, Ernest E. Moore, Massimo Sartelli, Dieter Weber, Chiara Robba

**Affiliations:** 1 Department of Anesthesia and Intensive Care, Parma University Hospital, Parma, Italy; 2 Department of General and Emergency Surgery, Bufalini Hospital, Cesena, Italy; 3 The Research Office, College of Medicine and Health Sciences, United Arab Emirates University, Al-Ain, United Arab Emirates; 4 Unit of General Surgery, San Matteo Hospital Pavia, University of Pavia, Pavia, Italy; 5 Department of Neurosurgery, 71541MedStar Georgetown University Hospital, Washington, DC USA; 6 Department of Neurosurgery, 8405MedStar Washington Hospital Center, Washington, DC USA; 7 Acute Care Surgery and Trauma Unit, Department of General Surgery, Hadassah Medical Center and Faculty of Medicine, Hebrew University of Jerusalem Kiriat Hadassah, Jerusalem, Israel; 8 Department of Traumatology, John Hunter Hospital, Hunter Medical Research Institute and University of Newcastle, Newcastle, NSW Australia; 9 Department of Neurosurgery, SS Antonio E Biagio E Cesare Arrigo Alessandria Hospital, Alessandria, Italy; 10 Scripps Clinic Medical Group, La Jolla, CA USA; 11 Inserm, U1216, CHU Grenoble Alpes, Grenoble Institut Neurosciences, Université Grenoble Alpes, Grenoble, France; 12 Department of Neurosurgery, Faculty of Medicine and Health, Örebro University, Örebro, Sweden; 13 Neuroradiology Unit, Azienda Ospedaliero-Universitaria of Parma, Parma, Italy; 14 Department of Neurological Surgery, University of Washington, Seattle, WA USA; 15 Department of Orthopedics and Sports Medicine, University of Washington, Seattle, WA USA; 16 Department of Global Health, University of Washington, Seattle, WA USA; 17 School of Medicine and Surgery, University of Milano-Bicocca, Monza, Italy; 18 Neuroscience Department, NeuroIntensive Care Unit, Hospital San Gerardo, ASST Monza, Monza, Italy; 19 Department of Emergency and Trauma Surgery, Pisa University Hospital, Pisa, Italy; 20 Riverside University Health System Medical Center, Loma Linda University School of Medicine, Riverside, CA USA; 21 Department of Anesthesia, Intensive Care and Prehospital Emergency, Ospedale Maggiore Carlo Alberto Pizzardi, Bologna, Italy; 22 Neuroradiology Unit, Department of Experimental and Clinical Biomedical Sciences, University of Florence, Florence, Italy; 23 Department of Neurosurgery, Neurosciences Centre and JPN Apex Trauma Centre, All India Institute of Medical Sciences, New Delhi, India; 24 Department of Trauma, San Antonio Military Medical Center and the U.S. Army Institute of Surgical Research, San Antonio, TX 78234 USA; 25 The Department of Defense Center of Excellence for Trauma, Joint Trauma System (JTS), JBSA Fort Sam Houston, San Antonio, TX 78234 USA; 26 Cleveland Clinic, 762 S. Cleveland-Massillon Rd, Akron, OH 44333 USA; 27 Neurological Intensive Care Unit, Department of Neurology, Medical University of Innsbruck, Innsbruck, Austria; 28 Department of Neurosurgery, Department of Clinical Neurosciences, University of Cambridge, Cambridge, UK; 29 Neurosurgery Unit, Department of Biomedical, Metabolic and Neural Sciences, University of Modena and Reggio Emilia, Azienda Ospedaliero-Universitaria di Modena, Modena, Italy; 30 National Institute for Health Research Global Health Research Group on Neurotrauma, University of Cambridge, Cambridge, UK; 31 Division of Neurosurgery, Department of Clinical Neurosciences, Addenbrooke’s Hospital,, University of Cambridge, Cambridge, UK; 32 Harborview Medical Center, University of Washington, Seattle, WA USA; 33 Division of Trauma and Acute Care Surgery, Los Angeles County + USC Medical Center, Los Angeles, CA USA; 34 Department of Intensive Care, University Hospitals Leuven, Louvain, Belgium; 35 Laboratory of Intensive Care Medicine, Katholieke Universiteit Leuven, Louvain, Belgium; 36 Department of Neurological Surgery, University of Pittsburgh Medical Center, Pittsburgh, PA USA; 37 Department of Anesthesia, Critical Care and Emergency, Spedali Civili University Hospital, Brescia, Italy; 38 Surgery Department, Section of Trauma Surgery, Hamad General Hospital (HGH), Doha, Qatar; 39 INUB-MEDITECH Research Group, Institute of Neurosciences, Universidad El Bosque, Bogotá, Colombia; 40 Department of Neurosurgery, Vall d’Hebron University Hospital, Universitat Autònoma de Barcelona, Barcelona, Spain; 41 Trauma Critical Care and Acute Care Surgery, Air Force Center for Sustainment of Trauma and Readiness Skills, University of Cincinnati Medical Center, Cincinnati, OH USA; 42 Department of Biomedical Sciences, Humanitas University, Pieve Emanuele, Milan, Italy; 43 Department of Neurosurgery, IRCCS Humanitas Research Hospital, Rozzano, Milan, Italy; 44 Department of Anesthesiology and Pain Medicine and Neurological Surgery, University of Washington, Seattle, WA USA; 45 Department of Critical Care Medicine, UPMC/University of Pittsburgh School of Medicine, Pittsburgh, PA USA; 46 College of Osteopathic Medicine, Rocky Vista University, Parker, CO USA; 47 Department of Intensive Care, Hôpital Universitaire de Bruxelles, Brussels, Belgium; 48 Department of Intensive Care and Hyperbaric Medicine, The Alfred, Melbourne, VIC 3004 Australia; 49 Department of Pathophysiology and Transplantation, University of Milan, Milan, Italy; 50 Department of Anesthesia, Critical Care and Emergency, Fondazione IRCCS Ca’ Granda Ospedale Maggiore Policlinico, Milan, Italy; 51 Anesthesia and Intensive Care Unit, AUSL Romagna, M. Bufalini Hospital, Cesena, Italy; 52 Healthcare Administration, Santa Maria Delle Croci Hospital, Ravenna, Italy; 53 Department of General, Digestive and Metabolic Minimally Invasive Surgery, Centre Hospitalier Intercommunal De Poissy/St Germain en Laye, Poissy, France; 54 Department of General Surgery, Rambam Health Care Campus, Haifa, Israel; 55 Department of Anesthesiology and Acute Care, Umberto I Hospital of Lugo, Ausl Della Romagna, Lugo, Italy; 56 Ernest E Moore Shock Trauma Center at Denver Health, University of Colorado, Denver, CO USA; 57 Department of Surgery, Macerata Hospital, Macerata, Italy; 58 Department of General Surgery, Royal Perth Hospital, The University of Western Australia, Perth, Australia; 59 Anesthesia and Intensive Care, San Martino Policlinico Hospital, IRCCS for Oncology and Neuroscience, Genoa, Italy; 60 Department of Surgical Sciences and Integrated Sciences, University of Genoa, Genoa, Italy

**Keywords:** Traumatic brain injury, Management, Transfer, Hub, Spoke

## Abstract

**Background:**

Severe traumatic brain-injured (TBI) patients should be primarily admitted to a hub trauma center (hospital with neurosurgical capabilities) to allow immediate delivery of appropriate care in a specialized environment. Sometimes, severe TBI patients are admitted to a spoke hospital (hospital without neurosurgical capabilities), and scarce data are available regarding the optimal management of severe isolated TBI patients who do not have immediate access to neurosurgical care.

**Methods:**

A multidisciplinary consensus panel composed of 41 physicians selected for their established clinical and scientific expertise in the acute management of TBI patients with different specializations (anesthesia/intensive care, neurocritical care, acute care surgery, neurosurgery and neuroradiology) was established. The consensus was endorsed by the World Society of Emergency Surgery, and a modified Delphi approach was adopted.

**Results:**

A total of 28 statements were proposed and discussed. Consensus was reached on 22 strong recommendations and 3 weak recommendations. In three cases, where consensus was not reached, no recommendation was provided.

**Conclusions:**

This consensus provides practical recommendations to support clinician’s decision making in the management of isolated severe TBI patients in centers without neurosurgical capabilities and during transfer to a hub center.

**Supplementary Information:**

The online version contains supplementary material available at 10.1186/s13017-022-00468-2.

## Background

Traumatic brain injury (TBI) is a leading cause of mortality and disability worldwide [1–4]. Severe TBI patients often require emergency neurosurgery (i.e., to remove post-traumatic mass lesions) and/or invasive neuromonitoring (i.e., to guide and personalize therapy) to improve mortality and neurological outcomes [1, 3, 5]. Considering the above, severe TBI patients should be primarily admitted to a hub trauma center (hospital with neurosurgical capabilities) to allow immediate delivery of appropriate care in a specialized environment [1, 3, 6–8]. Sometimes, severe TBI patients are admitted to a spoke hospital (hospital without neurosurgical capabilities) due to geographic or patient-related factors [9]. There is currently a paucity of available literature regarding the optimal management of severe isolated TBI patients at a spoke hospital [3]. The specific aim of this consensus is to provide recommendations on the early management of severe isolated TBI patients admitted to a spoke hospital and during the transfer to a hub hospital. Precisely, we refer to patients admitted to an urban spoke hospital without neurosurgical capabilities in a high-income country with the availability of an intensive care unit (ICU), operating room (OR) and computed tomography (CT) scan. In the case of severe TBI with extra-cranial lesions or admitted to hospitals with limited resources, readers can refer to several published articles/guidelines for initial management [10–14].

## Methods

The multidisciplinary consensus panel was composed of anesthesiologists/intensivists/neurointensivists (*n* = 13), neurosurgeons (*n* = 14), neuroradiologists (*n* = 2) and acute care surgeons (*n* = 13) with expertise in TBI care (see Additional file 1: Appendix 1). Following a non-systematic review of the literature, the steering committee (EP, CR and FC) identified the main domains to discuss and generated a list of questions to be addressed by the panel. Three subsequent online questionnaires were administered between July and September 2022. The initial list of statements (28) was formulated and distributed to the panelists 1 week prior to every Delphi round to allow modifications or additional statements. The modified interactive Delphi process was conducted using online tools. After a preliminary round, based on the initial answers and on comments/suggestions by the voting members, ambiguities and inconsistencies in the questionnaire were identified and corrected, generating a refined question set for subsequent voting rounds. We used an iterative approach; members were informed of the degree of consensus reached on the initial question round and asked to reconsider agreement or disagreement. Then, based on the answers collected in the third stage, statements for practical advice were proposed. The objective was to reach consensus, not necessarily unanimity.

The analysis of voting results was performed by a non-voting experienced methodologist (CR). A decision rule was predefined to ascertain the degree of consensus required to provide a recommendation. Statements were classified as strong recommendation, weak recommendation and no recommendation when > 85%, 75–85% and < 75% of votes were in favor, respectively.

In this consensus, we specifically refer to isolated severe TBI patients [Glasgow Coma Scale (GCS) score ≤ 8] admitted to a spoke non-rural hospital of a high-income country with the availability of: ICU, OR and whole-body CT scanner.

## Results

**Fig. 1:**
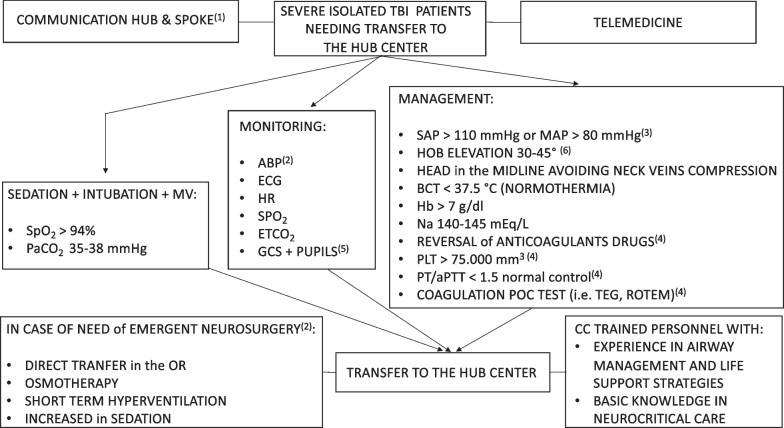
Consensus flowchart. ^(1)^Collaboration/communication (i.e., check for availability of ICU bed/OR, significant clinical deterioration during transfer, etc.) between different medical specialties (anesthesiology/intensive care/neurocritical care, neurosurgery, neuroradiology, trauma surgery, etc.). ^(2)^Patients with signs/elevated risk of herniation and need for emergent neurosurgery with brain CT scan already done in spoke hospital with neurosurgical consultation. ^(3)^In the case of invasive ABP monitoring, the arterial transducer should be zeroed at the level of the tragus. ^(4)^At risk of needing neurosurgery (including ICP monitoring). ^(5)^Serial examination in the spoke hospital and during transfer to the hub center to detect neuro-worsening. We recommend against the discontinuation of sedation to obtain a reliable neurological evaluation in patients with radiological signs of intracranial hypertension (i.e., midline shift, compression of the basal cisterns, sulcal effacement, etc.). In this scenario, only pupil examination, especially during the transfer, would be useful. ^(6)^Also during transfer. *Abbreviations* TBI, traumatic brain injury; ICP, intracranial pressure; ICU, intensive care unit; OR, operating room; CT, computed tomography; GCS, Glasgow coma scale; ABP, arterial blood pressure; ECG, electrocardiogram; HR, heart rate; SpO_2_, peripheral oxygen saturation; ETCO_2_, end-tidal carbon dioxide; MAP, mean arterial pressure; SAP, systolic arterial pressure; PLT, platelet; PT, prothrombin time; aPTT, activated partial thromboplastin time; POC, point-of-care; TEG, thromboelastography; ROTEM, rotational thromboelastometry; EEG, electroencephalogram; Hb, hemoglobin; PaCO_2_, arterial partial pressure of carbon dioxide; Na, sodium; CC, critical care

The consensus provided 25 recommendations (Table 1): 22 were strong recommendations, endorsed by more than 85% of participants, while 3 were weak recommendations, supported by 75–85%. The consensus flowchart is reported in Fig. 1. We were unable to reach consensus for 3 statements. The consensus recommendations are listed below with the percentage of agreement.

**Table 1:** List of consensus recommendations

N	Recommendation	Level
1	We recommend that all salvageable (i.e., patients who may recover, at least to some extent, with appropriate treatment) severe isolated TBI patients needing or at risk of needing neurosurgery [i.e., for surgical mass lesion and/or ICP monitoring] admitted to a spoke center should be rapidly transferred to a hub center after hemodynamic and respiratory stabilization	Strong recommendation
2	We recommend the utilization of a telemedicine service for rapid digital image transfer from the spoke to the hub center	Strong recommendation
3	We recommend, before and during transfer from the spoke to the hub center, a continuous and clear collaboration/communication (i.e., check for availability of ICU bed/OR, significant clinical deterioration during transfer, etc.) between different medical specialties (anesthesiology/intensive care/neurocritical care, neurosurgery, neuroradiology, trauma surgery, etc.)	Strong recommendation
4	We recommend sedation, intubation and mechanical ventilation for the transfer of all severe TBI patients	Strong recommendation
5	We recommend that the transfer of severe TBI patients should be performed by appropriately trained and certified critical care transport personnel with experience in advanced airway management/life support strategies and basic knowledge of neurocritical care (i.e., medical management of cerebral swelling, herniation)	Weak recommendation
6	We recommend that severe salvageable TBI patients with signs/elevated risk of herniation and need for neurosurgery (brain CT scan already done in spoke hospital with neurosurgical consultation) should be directly transported form the spoke center to the OR at the hub center	Strong recommendation
7	We recommend, in severe TBI patients needing transfer to the hub center, an invasive monitoring of ABP in addition to the standard cardiorespiratory monitoring (ECG, HR, SpO_2_ and ETCO_2_)	Weak recommendation
8	We recommend maintaining SAP > 110 mmHg or mean arterial pressure MAP > 80 mmHg* in severe isolated TBI patients *In the case of invasive ABP monitoring, the arterial transducer should be zeroed at the level of the tragus	Strong recommendation
9	We recommend maintaining PLT count > 75.000/mm^3^ in all salvageable severe TBI patients at risk of needing neurosurgery (including ICP monitoring)	Strong recommendation
10	We recommend maintaining PT/aPTT value < 1.5 the normal control in all salvageable severe TBI patients at risk of needing neurosurgery (including ICP monitoring)	Strong recommendation
11	We recommend early reversal of anticoagulant/antiplatelets agents, in all salvageable severe TBI patients at risk of needing neurosurgery (including ICP monitoring)	Strong recommendation
12	We recommend utilization of POC tests (i.e., TEG and ROTEM), if available, to optimize coagulation function in all salvageable severe TBI patients at risk of needing neurosurgery (including ICP monitoring)	Weak recommendation
13	We are unable to recommend the routine use of specific anti-seizure drugs in salvageable severe TBI patients presenting with seizure observed clinically and/or with EEG	No recommendation
14	We recommend performing serial neurologic evaluations (GCS + pupil examination) in the spoke center and during transfer to the hub center to detect neurologic deterioration in patients without signs of intracranial hypertension	Strong recommendation
15	We recommend against discontinuation of sedation to obtain a reliable neurological evaluation in patients with radiological signs of intracranial hypertension (i.e., midline shift, compression of the basal cisterns, sulcal effacement, etc.). In this scenario, only pupil examination, especially during the transfer, would be useful	Strong recommendation
16	We are unable to recommend use of brain ultrasonography (i.e., optic nerve sheath diameter, cerebral blood flow waveform analysis, etc.), in the presence of skilled operators, as a reliable screening non-invasive tool for detection of intracranial hypertension in the spoke center	No recommendation
17	We are unable to recommend use of automated pupillometry, if available, as a reliable screening non-invasive tool for detection of intracranial hypertension in the spoke center	No recommendation
18	We recommend that performance of brain ultrasonography and/or automated pupillometry, if utilized in the spoke center, should not significantly delay the patient’s transfer	Strong recommendation
19	We recommend that severe isolated TBI patients should be maintained with a head of the bed elevated at 30°–45° to facilitate brain venous drainage in the spoke center and during transfer to the hub center	Strong recommendation
20	We recommend that in severe TBI patients, the head should be maintained in the midline avoiding compression of the neck veins in the spoke center and during transfer to the hub center	Strong recommendation
21	We recommend avoiding core body temperature > 37.5 °C and to aim for normothermia in severe TBI patients	Strong recommendation
22	We recommend maintaining Hb level > 7 g/dl in severe TBI patients	Strong recommendation
23	We recommend maintaining SpO_2_ > 94% in severe TBI patients	Strong recommendation
24	We recommend maintaining a PaCO_2_ of 35–38 mmHg in severe TBI patients	Strong recommendation
25	We recommend maintaining a serum Na level of 140–145 mEq/l in severe TBI patients	Strong recommendation
26	We recommend osmotherapy as a therapeutic maneuver to be utilized in patients with signs of intracranial hypertension/brain herniation awaiting emergent neurosurgery	Strong recommendation
27	We recommend short-term hyperventilation as a therapeutic maneuver that should be utilized only in patients with signs of brain herniation awaiting emergent neurosurgery	Strong recommendation
28	We recommend an increase in sedation, while maintaining an acceptable ABP, as a therapeutic maneuver that should be utilized in the management of patients with signs of brain herniation awaiting emergent neurosurgery	Strong recommendation

TBI, traumatic brain injury; ICP, intracranial pressure; ICU, intensive care unit; OR, operating room; CT, computed tomography; GCS, Glasgow coma scale; ABP, arterial blood pressure; ECG, electrocardiogram; HR, heart rate; SpO_2_, peripheral oxygen saturation; ETCO_2_, end-tidal carbon dioxide; MAP, mean arterial pressure; SAP, systolic arterial pressure; PLT, platelet; PT, prothrombin time; aPTT, activated partial thromboplastin time; POC, point-of-care; TEG, thromboelastography; ROTEM, rotational thromboelastometry; EEG, electroencephalogram; Hb, hemoglobin; PaCO_2_, arterial partial pressure of carbon dioxide; Na, sodium

### Recommendation 1

We recommend that all salvageable (i.e., patients who may recover, at least to some extent, with appropriate treatment) severe isolated TBI patients needing or at risk of needing neurosurgery [i.e., for surgical mass lesion and/or intracranial pressure (ICP) monitoring] admitted to a spoke center should be rapidly transferred to a hub center after hemodynamic and respiratory stabilization (agreement %: 97.6, *strong* recommendation).

### Recommendation 2

We recommend the utilization of a telemedicine service for rapid digital image transfer from the spoke to the hub center (agreement %: 92.7, *strong* recommendation).

### Recommendation 3

We recommend, before and during transfer from the spoke to the hub center, a continuous and clear collaboration/communication (i.e., check for availability of ICU bed/OR, significant clinical deterioration during transfer, etc.) between different medical specialties (anesthesiology/intensive care/neurocritical care, neurosurgery, neuroradiology, trauma surgery, etc.) (agreement %: 92.7, *strong* recommendation).

### Recommendation 4

We recommend sedation, intubation and mechanical ventilation for the transfer of all severe TBI patients (agreement %: 95, *strong* recommendation).

### Recommendation 5

We recommend that the transfer of severe TBI patients should be performed by appropriately trained and certified critical care transport personnel with experience in advanced airway management/life support strategies and basic knowledge of neurocritical care (i.e., medical management of cerebral swelling, herniation) (agreement %: 80.5, *weak* recommendation).

### Recommendation 6

We recommend that severe salvageable TBI patients with signs/elevated risk of herniation and need for neurosurgery (brain CT scan already done in spoke hospital with neurosurgical consultation) should be directly transported form the spoke center to the OR at the hub center (agreement %: 85.1, *strong* recommendation).

### Recommendation 7

We recommend, in severe TBI patients needing transfer to the hub center, an invasive monitoring of arterial blood pressure (ABP) in addition to the standard cardiorespiratory monitoring [electrocardiogram (ECG), heart rate (HR), peripheral oxygen saturation (SpO_2_) and end-tidal carbon dioxide (ETCO_2_)] (agreement %: 82.9, *weak* recommendation).

### Recommendation 8

We recommend maintaining systolic arterial pressure (SAP) > 110 mmHg or mean arterial pressure (MAP) > 80 mmHg* in severe isolated TBI patients (agreement %: 90, *strong* recommendation).

* In the case of invasive ABP monitoring, the arterial transducer should be zeroed at the level of the tragus.

### Recommendation 9

We recommend maintaining platelet (PLT) count > 75.000/mm^3^ in all salvageable severe TBI patients at risk of needing neurosurgery (including ICP monitoring) (agreement %: 92, *strong* recommendation).

### Recommendation 10

We recommend maintaining prothrombin time (PT)/activated partial thromboplastin time (aPTT) value < 1.5 the normal control in all salvageable severe TBI patients at risk of needing neurosurgery (including ICP monitoring) (agreement %: 90, *strong* recommendation).

### Recommendation 11

We recommend early reversal of anticoagulant/antiplatelets agents, in all salvageable severe TBI patients at risk of needing neurosurgery (including ICP monitoring) (agreement %: 90, *strong* recommendation).

### Recommendation 12

We recommend utilization of point-of-care (POC) tests [i.e., thromboelastography (TEG) and rotational thromboelastometry (ROTEM)], if available, to optimize coagulation function in all salvageable severe TBI patients at risk of needing neurosurgery (including ICP monitoring) (agreement %: 75.6, weak recommendation).

### Recommendation 13

We are unable to recommend the routine use of specific anti-seizure drugs in salvageable severe TBI patients presenting with seizure observed clinically and/or with electroencephalogram (EEG) (agreement %: 65.9, *no* recommendation).

### Recommendation 14

We recommend performing serial neurologic evaluations (GCS + pupil examination) in the spoke center and during transfer to the hub center to detect neurologic deterioration in patients without signs of intracranial hypertension (agreement %: 95, *strong* recommendation).

### Recommendation 15

We recommend against discontinuation of sedation to obtain a reliable neurological evaluation in patients with radiological signs of intracranial hypertension (i.e., midline shift, compression of the basal cisterns, sulcal effacement, etc.). In this scenario, only pupil examination, especially during the transfer, would be useful (agreement %: 95, *strong* recommendation).

### Recommendation 16

We are unable to recommend use of brain ultrasonography (i.e., optic nerve sheath diameter, cerebral blood flow waveform analysis, etc.), in the presence of skilled operators, as a reliable screening non-invasive tool for detection of intracranial hypertension in the spoke center (agreement % 61, *no* recommendation).

### Recommendation 17

We are unable to recommend use of automated pupillometry, if available, as a reliable screening non-invasive tool for detection of intracranial hypertension in the spoke center (agreement %: 63, *no* recommendation).

### Recommendation 18

We recommend that performance of brain ultrasonography and/or automated pupillometry, if utilized in the spoke center, should not significantly delay the patient’s transfer (agreement %: 90, *strong* recommendation).

### Recommendation 19

We recommend that severe isolated TBI patients should be maintained with a head of the bed elevated at 30°–45° to facilitate brain venous drainage in the spoke center and during transfer to the hub center (agreement %: 92.7, *strong* recommendation).

### Recommendation 20

We recommend that, in severe TBI patients, the head should be maintained in the midline avoiding compression of the neck veins in the spoke center and during transfer to the hub center (agreement %: 97.7, *strong* recommendation).

### Recommendation 21

We recommend avoiding core body temperature > 37.5 °C and to aim for normothermia in severe TBI patients (agreement %: 95, *strong* recommendation).

### Recommendation 22

We recommend maintaining hemoglobin (Hb) level > 7 g/dl in severe TBI patients (agreement %: 95, *strong* recommendation).

### Recommendation 23

We recommend maintaining SpO_2_ > 94% in severe TBI patients (agreement %: 87.8, *strong* recommendation).

### Recommendation 24

We recommend maintaining an arterial partial pressure of carbon dioxide (PaCO_2_) of 35–38 mmHg in severe TBI patients (agreement %: 90, *strong* recommendation).

### Recommendation 25

We recommend maintaining a serum sodium (Na) level of 140–145 mEq/l in severe TBI patients (agreement %: 90, *strong* recommendation).

### Recommendation 26

We recommend osmotherapy as a therapeutic maneuver to be utilized in patients with signs of intracranial hypertension/brain herniation awaiting emergent neurosurgery (agreement %: 95, *strong* recommendation).

### Recommendation 27

We recommend short-term hyperventilation as a therapeutic maneuver that should be utilized only in patients with signs of brain herniation awaiting emergent neurosurgery (agreement %: 90, *strong* recommendation).

### Recommendation 28

We recommend an increase in sedation, while maintaining an acceptable ABP, as a therapeutic maneuver that should be utilized in the management of patients with signs of brain herniation awaiting emergent neurosurgery (agreement %: 95, *strong* recommendation).

## Discussion

### Transfer to the HUB center

All salvageable severe isolated TBI patients, needing or at risk of needing neurosurgery (*i.e.,* for surgical mass lesion and/or ICP monitoring) [15–19], should be transported to a hub center (hospital with neurosurgical capabilities). This is important not only for the surgical aspect but also to facilitate appropriate care in a specialized environment [1, 3, 6–8]. Moreover, a recent systematic review and meta-analysis showed that neurocritical care services are associated with improved survival and functional outcomes in critically ill adults with brain injury [20].

The transfer, as recommended also by recent guidelines [21], should be performed after cardiorespiratory stabilization. Furthermore, before and during transfer from the spoke to the hub center, a continuous and clear collaboration/communication (i.e., check for availability of ICU bed/OR, significant clinical deterioration during transfer, etc.) should occur between different medical specialties (anesthesiology/intensive care/neurocritical care, neurosurgery, neuroradiology, trauma surgery, etc.). The above-mentioned aspects, in addition to be in accordance with published guidelines [21], are of paramount importance to ensure “high-quality” TBI perioperative care [3]. In particular, severe salvageable TBI patients with signs/elevated risk of herniation and need for neurosurgery (brain CT scan already done in spoke hospital with neurosurgical consultation) could benefit from direct transport from the spoke center to the OR in the hub center. This requires not only an excellent coordination between all involved medical specialties, but review of CT scans and laboratories prior to the patient arrival; this concept is similar to what is applied in some trauma centers in the treatment of severely injured patients at increased risk for requiring lifesaving interventions or emergency surgery [22]. Some panelists suggested that patients requiring urgent surgery should be transferred irrespective of the availability of an ICU bed. This is an interesting and debated point. A TBI patient needing evacuation of a critical mass lesion could be transferred to the nearest neurosurgical unit for an operation regardless of ICU bed availability; this problem could be arranged in the post-surgical phase.

Telemedicine, allowing the transfer of radiological images by a web-based software, facilitates neurosurgical consultation between hospitals and, preventing unnecessary transfers, is life/time-saving and cost-effective [23, 24]. Telemedicine should be encouraged in this setting as has been already done for aneurysmal subarachnoid hemorrhage (aSAH) patients [25]. Severe TBI patients can also deteriorate at any time during the transfer (i.e., neuro-worsening, hemodynamic instability, etc.). As also suggested in other guidelines [21], these patients should be accompanied during the transport by a physician with expertise in airway management, life support strategies and basic knowledge of neurocritical care. Some panelists pointed out that worldwide there can be different systems regarding the transfer of critical care patients (i.e., paramedics). Regarding these possible organizational differences, the transfer should be carried out by appropriately trained and certified critical care transport personnel to ensure adequate quality of care. Practical protocols between hub and spoke hospitals should be encouraged to facilitate the transfer. In this regard, a prepared and shared checklist could be helpful.

Considering the above-mentioned points, adequate cardiorespiratory monitoring (ECG, HR, ABP, SpO_2_ and ETCO_2_) seems to be fundamental for the safety of the patients during the transfer [21]. Invasive ABP monitoring, being accurate and continuous, is preferable (especially in unstable severe TBI patients). However, placement of an arterial line should not excessively delay the patient transfer, and non-invasive ABP (NIABP) monitoring should be considered as a valid alternative in cases of difficult arterial access.

### Airway, respiratory, hemodynamic, electrolytes and temperature management

Severe isolated TBI patients require tracheal intubation (to protect the airway) and mechanical ventilation (to optimize gas exchange) [26]. Tracheal intubation needs to be performed carefully with adequate analgesia and sedation to avoid arterial desaturation, increase in ABP exacerbating pre-existing intracranial hematoma or severe hypotension with associated cerebral hypoperfusion [21]. Some panelists suggested the utilization of drugs with a short half-life and easily titratable to allow a reliable neurological examination.

Episodes of SpO_2_ < 90%, being associated with increased mortality and worse neurological outcome in TBI, should be avoided [26, 27]. Cerebral perfusion is influenced by PaCO_2_ level, and ventilation should be adjusted to avoid hypo/hypercapnia [26, 28]. According to recent consensus conferences [26, 29], we recommend maintaining SpO_2_ > 94% and a PaCO_2_ of 35–38 mmHg. We are aware that optimal respiratory values have yet to be determined in this setting. Moreover, the absence of invasive neuromonitoring (generally available in the referral center) prevents the individualization (personalization) of care. ETCO_2_ values should be adapted frequently with data from arterial blood gas analysis.

Arterial hypotension (SAP < 90 mmHg), similar to hypoxia, has been associated with worse neurological outcomes in TBI [30]. In this regard, the Brain Trauma Foundation (BTF) guidelines recommend maintaining SAP at 100 mmHg for patients 50–69 years old or 110 mmHg or above for patients 15–49 or > 70 years old [17]. The European guidelines regarding the management of major hemorrhage and coagulopathy in polytrauma patients recommend maintaining MAP ≥ 80 mmHg in the case of severe TBI (grade 1C) [10]. Considering the above, we recommend maintaining SAP > 110 mmHg or MAP > 80 mmHg in severe isolated TBI patients. In the case of invasive ABP monitoring, we suggest that the arterial transducer should be zeroed at the level of the tragus according to the joint position statement by the councils of the Neuroanaesthesia and Critical Care Society of Great Britain and Ireland (NACCS) and the Society of British Neurological Surgeons (SBNS) [31]. About this, some panelists have expressed concerns. Specifically, zeroing the transducer at the level of the tragus and maintaining head of the bed elevated at 30°–45° (to facilitate brain venous drainage) could result in higher ABP values respect to zeroing made at the level of the heart. This is an interesting aspect requiring further research.

Pending the results of ongoing trials [“Transfusion Strategies in Acute Brain-Injured Patients (TRAIN)” study (NCT02968654) and the “HEMOglobin transfusion threshold in Traumatic brain Injury OptimizatioN: The HEMOTION Trial” (NCT03260478)] and according to guidelines/consensus [10, 29], we recommend maintaining Hb level > 7 g/dl in severe isolated TBI patients.

Hyponatremia can be detrimental for TBI patients at risk of intracranial hypertension and should be avoided [29, 32, 33]. In this regard, we recommend maintaining serum Na in the upper limit of the normal range.

Fever is a dangerous secondary insult for the injured brain associated with worse neurological outcome [34]. The optimal threshold to start antipyretics therapy in TBI has not yet been established [35]. Considering the gradient between core and brain temperature (brain > core) [36], we recommend avoiding core body temperature > 37.5 °C and to aim for normothermia.

### Coagulation management

Coagulopathy, associated with TBI or with previously administered drugs, is frequently encountered after head injury, and the consequent progression of intracranial mass lesions is often associated with unfavorable neurological outcome [37–40]. The rapid correction of trauma- or medication-induced coagulopathy is very important, especially for patients needing urgent neurosurgical procedures. Whether the management of hemostatic abnormalities after TBI can protect against secondary brain injury and improve neurological outcomes remains elusive [41]; no specific guidelines regarding coagulation management in TBI patients have been published to date. Basic coagulation parameters suggested for neurosurgery are: PLT count > 75.000 or 100.000/mm^3^ and PT/aPTT < 1.5 the normal control values [10, 42, 43]. The utilization of POC tests, such as TEG and ROTEM, may be useful to personalize therapy in this setting and in the case of utilization of antiplatelets drugs and/or direct oral anticoagulants (DOACs) [44]. Our recommendations are in agreement with the above. We are aware that POC tests are not available worldwide. Their use can be considered, but without significantly delay the transfer. Some panelists suggested a PLT count > 100.000/mm^3^ compared with > 75.000 mm^3^; in this regard, the optimal cut-off for TBI patients at risk of needing neurosurgery has yet to be established. According to some panelists, reversal of antiplatelets/anticoagulants drugs should be started immediately prior to neurosurgery and for others, optimization of coagulation should not delay the transfer too much and could even be done “en route.” We suggest readers to refer to the “Guideline for Reversal of Antithrombotics in Intracranial Hemorrhage” of the Neurocritical Care Society (NCS) and the Society of Critical Care Medicine (SCCM) published in 2016 with the aim to provide timely and evidence-based reversal strategies for the care of patients with antithrombotic-associated intracranial hemorrhage [44].

### Neuromonitoring

ICP monitoring is usually not available in spoke centers and during transfer to hub centers. However, different tools can help us to estimate the risk of intracranial hypertension. A basic neurological evaluation, including GCS and pupil examination (size and reaction), is very helpful to identify neurological deterioration associated with an increase of ICP [5, 45]. The motor response is the most easily evaluable component of the GCS (especially in conditions where verbal response and eye opening can be difficult to obtain, as in the case of tracheal intubation, facial injuries, etc.) [5, 45]. Pupillary shape/diameter and reactivity to light should be carefully evaluated [5, 46, 47]. These evaluations are essential before tracheal intubation and sedation. However, discontinuation of sedation to obtain a reliable neurological evaluation can be dangerous in patients with radiological signs of increased ICP [48]. Although not specific, certain CT radiological signs are suggestive of intracranial hypertension such as the compression of the basal cisterns, midline shift and sulcal effacement [49, 50].

Brain ultrasonography, when performed by a skilled operator, can estimate intracranial hypertension by the evaluation of optic nerve sheath diameter (ONSD), pulsatility index (PI), etc. [51].

Automated pupillometry accurately measures the pupil size and several dynamic variables such as pupillary constriction, latency and constriction/dilation velocity [51]. The integration of the latter into an algorithm provides the Neurological Pupil index (NPi—values 0–5; pathological < 3) [52]. Episodes of raised ICP are associated with a concomitant decrease of the NPi [52]. Considering the above, we recommend performing serial neurologic evaluations (GCS + pupil examination) in the spoke center and during transfer to the hub center to detect neuro-worsening in the absence of radiological signs of intracranial hypertension (i.e., midline shift, compression of the basal cisterns, sulcal effacement, etc.). In this scenario, only pupillary evaluation (shape/diameter and reactivity to light), especially during transfer, could be useful. We were unable to reach a consensus on the utilization of brain ultrasonography and automated pupillometry as a screening non-invasive tool for detection of intracranial hypertension in the spoke center. This could be related, at the moment, to the absence of robust data deriving from well-powered studies on this topic. The possible utilization of brain ultrasonography and automated pupillometry, by experienced operators, should not significantly delay the patient’s transfer.

### Brain-focused therapy

The maintenance of the head of a severe isolated TBI patients in the midline, avoiding compression of the neck veins, and with a bed elevated at 30°–45° to facilitate brain venous drainage are basic maneuvers in the neurocritical care setting [29]. These should always be applied with adequate spine precautions. Some panelists emphasized as some patients may require a lower bed elevation; this, considering what has been said previously for the zeroing of the arterial transducer in the case of invasive ABP monitoring, could lead to a reduced use of vasopressors for maintaining the blood pressure target.

Osmotherapy (i.e., mannitol or hypertonic saline) is effective in the rapid control of ICP through a reduction in blood viscosity and an increase in plasma osmolarity [53]. Currently, adequately powered randomized controlled studies clearly showing the superiority of mannitol compared to hypertonic saline are lacking. Considering the diuretic effect of mannitol, hypertonic saline is a potentially reasonable choice in cases of hypovolemia [29]. Moreover, the early utilization of mannitol, but not hypertonic saline, seems to be associated with increased incidence of acute kidney injury [54].

Hypocapnia associated with hyperventilation results in cerebral vasoconstriction with a reduction in cerebral blood flow (CBF), cerebral blood volume and consequently ICP [28]. This temporary effect is associated with the risk of development of cerebral ischemia [55]. Profound hypocapnia is not recommended as a prophylactic maneuver but could be utilized briefly for patients awaiting emergent neurosurgery [17, 26].

Metabolic suppression with sedatives can be useful in the control of intracranial hypertension but can increase the risk of hypotension [56, 57]. The reduction in blood pressure, observed in this scenario, should be aggressively corrected. In this regard, ketamine could be a useful option, but more data are necessary to confirm this [58].

Considering the above, in patients with signs of intracranial hypertension/brain herniation awaiting emergent neurosurgery, we recommend osmotherapy, short-term hyperventilation and an increase in sedation (ensuring an acceptable ABP).

According to BTF guidelines [17], prophylactic phenytoin or valproate are not recommended for preventing late post-traumatic seizures (PTS) and phenytoin is recommended to decrease the incidence of early PTS (within 7 days of injury), when the overall benefit is thought to outweigh the complications associated with such treatment (early PTS have not been associated with worse outcomes). Considering the above and awaiting the results of the ongoing “Management of Seizure after Traumatic Brain Injury” (MAST) trial (NCT04573803), we were unable to provide any recommendations regarding seizure prophylaxis in severe isolated TBI patients.

### Notes on the use of the current consensus

The aim of this consensus is to support clinicians’ decision making in the early management of isolated severe TBI patients admitted to a hospital without neurosurgical capabilities. The included statements are created to assist the physician’s clinical judgment, which is necessary to provide appropriate (personalized) therapy. Considering the lack of high-quality studies in this setting, we adopted a modified Delphi approach involving experts from different countries worldwide; this approach is less rigorous than evidence-based guidelines. However, we think that our methodology can provide useful recommendations for these challenging clinical scenarios. The practice guidelines promulgated in this work do not represent a standard of practice. They are suggested plans of care, based on the best available evidence and the consensus of experts, but they do not exclude other approaches as being within the standard of practice. Ultimately, responsibility for the results of treatment rests with those who are directly engaged therein, and not with the consensus group.

## Conclusions

Future studies should be encouraged to improve clinical outcomes for patients with severe TBI who do not have immediate access to neurosurgical care. This international multidisciplinary consensus conference was aimed to provide practical recommendations to deliver the best early possible care of severe isolated TBI patients admitted to a spoke center (without neurosurgical capabilities) and during the transfer to the hub center (with neurosurgical capabilities).

## Supplementary Information

**Additional file 1. Appendix 1.** Consensus participants.

## Data Availability

The datasets used and/or analyzed during the current study are available from the corresponding author on reasonable request.

## Abbreviations

TBI: Traumatic brain injury
WSES: World Society of Emergency Surgery
ICU: Intensive care unit
OR: Operating room
CT: Computed tomography
GCS: Glasgow Coma Scale
ICP: Intracranial pressure
ABP: Arterial blood pressure
ECG: Electrocardiogram
HR: Heart rate
SpO_2_: Peripheral oxygen saturation
ETCO_2_: End-tidal carbon dioxide
SAP: Systolic arterial pressure
MAP: Mean arterial pressure
PLT: Platelet
PT: Prothrombin time
aPTT: Activated partial thromboplastin time
POC: Point-of-care
TEG: Thromboelastography
ROTEM: Rotational thromboelastometry
EEG: Electroencephalogram
Hb: Hemoglobin
PaCO_2_: Arterial partial pressure of carbon dioxide
Na: Sodium
aSAH: Aneurysmal subarachnoid hemorrhage
NIABP: Non-invasive arterial blood pressure
BTF: Brain Trauma Foundation
NACCS: Neuroanaesthesia and Critical Care Society of Great Britain and Ireland
SBNS: Society of British Neurological Surgeons
TRAIN: Transfusion Strategies in Acute Brain-Injured Patients
DOACs: Direct oral anticoagulants
NCS: Neurocritical Care Society
SCCM: Society of Critical Care Medicine
ONSD: Optic nerve sheath diameter
PI: Pulsatility index
NPi: Neurological Pupil index
CBF: Cerebral blood flow
PTS: Post-traumatic seizures
MAST: Management of Seizure after Traumatic Brain Injury
