# Correction: Early management of isolated severe traumatic brain injury patients in a hospital without neurosurgical capabilities: a consensus and clinical recommendations of the World Society of Emergency Surgery (WSES)

**DOI:** 10.1186/s13017-023-00489-5

**Published:** 2023-04-06

**Authors:** Edoardo Picetti, Fausto Catena, Fikri Abu-Zidan, Luca Ansaloni, Rocco A. Armonda, Miklosh Bala, Zsolt J. Balogh, Alessandro Bertuccio, Walt L. Biffl, Pierre Bouzat, Andras Buki, Davide Cerasti, Randall M. Chesnut, Giuseppe Citerio, Federico Coccolini, Raul Coimbra, Carlo Coniglio, Enrico Fainardi, Deepak Gupta, Jennifer M. Gurney, Gregory W. J. Hawryluk, Raimund Helbok, Peter J. A. Hutchinson, Corrado Iaccarino, Angelos Kolias, Ronald W. Maier, Matthew J. Martin, Geert Meyfroidt, David O. Okonkwo, Frank Rasulo, Sandro Rizoli, Andres Rubiano, Juan Sahuquillo, Valerie G. Sams, Franco Servadei, Deepak Sharma, Lori Shutter, Philip F. Stahel, Fabio S. Taccone, Andrew Udy, Tommaso Zoerle, Vanni Agnoletti, Francesca Bravi, Belinda De Simone, Yoram Kluger, Costanza Martino, Ernest E. Moore, Massimo Sartelli, Dieter Weber, Chiara Robba

**Affiliations:** 1 Department of Anesthesia and Intensive Care, Parma University Hospital, Parma, Italy; 2 Department of General and Emergency Surgery, Bufalini Hospital, Cesena, Italy; 3 The Research Office, College of Medicine and Health Sciences, United Arab Emirates University, Al-Ain, United Arab Emirates; 4 Unit of General Surgery, San Matteo Hospital Pavia, University of Pavia, Pavia, Italy; 5 Department of Neurosurgery, 71541MedStar Georgetown University Hospital, Washington, DC USA; 6 Department of Neurosurgery, 8405MedStar Washington Hospital Center, Washington, DC USA; 7 Acute Care Surgery and Trauma Unit, Department of General Surgery, Hadassah Medical Center and Faculty of Medicine, Hebrew University of Jerusalem Kiriat Hadassah, Jerusalem, Israel; 8 Department of Traumatology, John Hunter Hospital, Hunter Medical Research Institute and University of Newcastle, Newcastle, NSW Australia; 9 Department of Neurosurgery, SS Antonio E Biagio E Cesare Arrigo Alessandria Hospital, Alessandria, Italy; 10 Scripps Clinic Medical Group, La Jolla, CA USA; 11 Inserm, U1216, CHU Grenoble Alpes, Grenoble Institut Neurosciences, Université Grenoble Alpes, Grenoble, France; 12 Department of Neurosurgery, Faculty of Medicine and Health, Örebro University, Örebro, Sweden; 13 Neuroradiology Unit, Azienda Ospedaliero-Universitaria of Parma, Parma, Italy; 14 Department of Neurological Surgery, University of Washington, Seattle, WA USA; 15 Department of Orthopedics and Sports Medicine, University of Washington, Seattle, WA USA; 16 Department of Global Health, University of Washington, Seattle, WA USA; 17 School of Medicine and Surgery, University of Milano-Bicocca, Monza, Italy; 18 Neuroscience Department, NeuroIntensive Care Unit, Hospital San Gerardo, ASST Monza, Monza, Italy; 19 Department of Emergency and Trauma Surgery, Pisa University Hospital, Pisa, Italy; 20 Riverside University Health System Medical Center, Loma Linda University School of Medicine, Riverside, CA USA; 21 Department of Anesthesia, Intensive Care and Prehospital Emergency, Ospedale Maggiore Carlo Alberto Pizzardi, Bologna, Italy; 22 Neuroradiology Unit, Department of Experimental and Clinical Biomedical Sciences, University of Florence, Florence, Italy; 23 Department of Neurosurgery, Neurosciences Centre and JPN Apex Trauma Centre, All India Institute of Medical Sciences, New Delhi, India; 24 Department of Trauma, San Antonio Military Medical Center and the U.S. Army Institute of Surgical Research, San Antonio, TX 78234 USA; 25 The Department of Defense Center of Excellence for Trauma, Joint Trauma System (JTS), JBSA Fort Sam Houston, San Antonio, TX 78234 USA; 26 Cleveland Clinic, 762 S. Cleveland-Massillon Rd, Akron, OH 44333 USA; 27 Neurological Intensive Care Unit, Department of Neurology, Medical University of Innsbruck, Innsbruck, Austria; 28 Department of Neurosurgery, Department of Clinical Neurosciences, University of Cambridge, Cambridge, UK; 29 Neurosurgery Unit, Department of Biomedical, Metabolic and Neural Sciences, University of Modena and Reggio Emilia, Azienda Ospedaliero-Universitaria di Modena, Modena, Italy; 30 National Institute for Health Research Global Health Research Group on Neurotrauma, University of Cambridge, Cambridge, UK; 31 Division of Neurosurgery, Department of Clinical Neurosciences, Addenbrooke’s Hospital,, University of Cambridge, Cambridge, UK; 32 Harborview Medical Center, University of Washington, Seattle, WA USA; 33 Division of Trauma and Acute Care Surgery, Los Angeles County + USC Medical Center, Los Angeles, CA USA; 34 Department of Intensive Care, University Hospitals Leuven, Louvain, Belgium; 35 Laboratory of Intensive Care Medicine, Katholieke Universiteit Leuven, Louvain, Belgium; 36 Department of Neurological Surgery, University of Pittsburgh Medical Center, Pittsburgh, PA USA; 37 Department of Anesthesia, Critical Care and Emergency, Spedali Civili University Hospital, Brescia, Italy; 38 Surgery Department, Section of Trauma Surgery, Hamad General Hospital (HGH), Doha, Qatar; 39 INUB-MEDITECH Research Group, Institute of Neurosciences, Universidad El Bosque, Bogotá, Colombia; 40 Department of Neurosurgery, Vall d’Hebron University Hospital, Universitat Autònoma de Barcelona, Barcelona, Spain; 41 Trauma Critical Care and Acute Care Surgery, Air Force Center for Sustainment of Trauma and Readiness Skills, University of Cincinnati Medical Center, Cincinnati, OH USA; 42 Department of Biomedical Sciences, Humanitas University, Pieve Emanuele, Milan, Italy; 43 Department of Neurosurgery, IRCCS Humanitas Research Hospital, Rozzano, Milan, Italy; 44 Department of Anesthesiology and Pain Medicine and Neurological Surgery, University of Washington, Seattle, WA USA; 45 Department of Critical Care Medicine, UPMC/University of Pittsburgh School of Medicine, Pittsburgh, PA USA; 46 College of Osteopathic Medicine, Rocky Vista University, Parker, CO USA; 47 Department of Intensive Care, Hôpital Universitaire de Bruxelles, Brussels, Belgium; 48 Department of Intensive Care and Hyperbaric Medicine, The Alfred, Melbourne, VIC 3004 Australia; 49 Department of Pathophysiology and Transplantation, University of Milan, Milan, Italy; 50 Department of Anesthesia, Critical Care and Emergency, Fondazione IRCCS Ca’ Granda Ospedale Maggiore Policlinico, Milan, Italy; 51 Anesthesia and Intensive Care Unit, AUSL Romagna, M. Bufalini Hospital, Cesena, Italy; 52 Healthcare Administration, Santa Maria Delle Croci Hospital, Ravenna, Italy; 53 Department of General, Digestive and Metabolic Minimally Invasive Surgery, Centre Hospitalier Intercommunal De Poissy/St Germain en Laye, Poissy, France; 54 Department of General Surgery, Rambam Health Care Campus, Haifa, Israel; 55 Department of Anesthesiology and Acute Care, Umberto I Hospital of Lugo, Ausl Della Romagna, Lugo, Italy; 56 Ernest E Moore Shock Trauma Center at Denver Health, University of Colorado, Denver, CO USA; 57 Department of Surgery, Macerata Hospital, Macerata, Italy; 58 Department of General Surgery, Royal Perth Hospital, The University of Western Australia, Perth, Australia; 59 Anesthesia and Intensive Care, San Martino Policlinico Hospital, IRCCS for Oncology and Neuroscience, Genoa, Italy; 60 Department of Surgical Sciences and Integrated Sciences, University of Genoa, Genoa, Italy

**Correction to: World Journal of Emergency Surgery (2023) 18:5** 10.1186/s13017-022-00468-2

Following publication of the original article [1], in this article the author name Gregory W. J. Hawryluk was incorrectly written as Gregory W. J. Hawrylux.

The original article has been corrected.
