# Supplementary material for: Early management of isolated severe traumatic brain injury patients in a hospital without neurosurgical capabilities: a consensus and clinical recommendations of the World Society of Emergency Surgery (WSES)
Source: World J Emerg Surg. 2023 Jan 9;18:5. doi: 10.1186/s13017-022-00468-2 (PMC9830860; doi:10.1186/s13017-022-00468-2)
Supplement: Supplementary file 1 — Additional file 1. Appendix 1. Consensus participants. [file 13017_2022_468_MOESM1_ESM.docx]

**Appendix 1.** Consensus participants.

1. Fikri Abu-Zidan
2. Luca Ansaloni
3. Rocco A Armonda
4. Miklosh Bala
5. Zsolt J Balogh
6. Alessandro Bertuccio
7. Walt L Biffl
8. Pierre Bouzat
9. Andras Buki
10. Fausto Catena
11. Davide Cerasti
12. Randall M Chesnut
13. Giuseppe Citerio
14. Federico Coccolini
15. Raul Coimbra
16. Carlo Coniglio
17. Enrico Fainardi
18. Deepak Gupta^21^
19. Jennifer M Gurney
20. Gregory WJ Hawrylux
21. Raimund Helbok
22. Peter JA Hutchinson
23. Corrado Iaccarino
24. Angelos Kolias
25. Ronald W Maier
26. Matthew J Martin
27. Geert Meyfroidt
28. David O Okonkwo
29. Frank Rasulo
30. Sandro Rizoli
31. Chiara Robba
32. Andres Rubiano
33. Juan Sahuquillo
34. Valerie G Sams
35. Franco Servadei
36. Deepak Sharma
37. Lori Shutter
38. Philip F Stahel
39. Fabio S Taccone
40. Andrew Udy
41. Tommaso Zoerle
